# Minilap-assisted modified single-incision *vs.* traditional single-incision laparoscopic cholecystectomy: a retrospective cohort study on instrument conflict and operative efficiency

**DOI:** 10.7717/peerj.20807

**Published:** 2026-03-10

**Authors:** Zheng Zhou, Xianqing Chen, Xiyan Zheng, Zhiqun Lin, Fei Du, Maoyun Xie, Xianjie Shi

**Affiliations:** Department of Hepatobiliary Pancreatic Surgery, The Eighth Affiliated Hospital of Sun Yat-Sen University, Shenzhen, China

**Keywords:** Minilapz, Single-incision, Laparoscopic cholecystectomy, Instrument conflict, Operative efficienc

## Abstract

**Background:**

The single-incision laparoscopic cholecystectomy (SILC) achieves a scarless appearance through a single umbilical approach. However, the restricted surgical field of view, frequent instrument collisions limit its clinical applicability. This study introduces minilap-assisted modified single-incision laparoscopic cholecystectomy (MSILC), incorporating two adjacent trocars at the umbilicus, combined with a 2-mm auxiliary hole created beneath the right costal margin, to mitigate these limitations while preserving aesthetic outcomes.

**Objective:**

To explore the clinical value of MSILC compared with traditional SILC (TSILC) in reducing instrument conflict, shortening operation time, improving operator efficiency and cosmetic effects, and providing a basis for the selection of surgical procedures for patients with high body mass index (BMI).

**Methods:**

A total of 51 patients with benign gallbladder diseases who underwent MSILC or TSILC between December 2021 and April 2025 at our institution were retrospectively enrolled and categorized into the MSILC (*n* = 25) or TSILC (*n* = 26) groups, respectively. For MSILC, a double-channel trocar was deployed at the umbilicus, combined with a 2-mm auxiliary hole layout placed beneath the right costal margin; TSILC comprised a single umbilical incision. The primary endpoint was the number of intraoperative instrument conflicts, while the secondary endpoints included operation time, Surgical difficulty score, operator comfort, and cosmetic satisfaction regarding the incision.

**Results:**

In the MSILC group, the number of instrument conflicts was reduced by 38.5% (median 16 *vs*. 26, *P* < 0.001), while the operator comfort score improved by 50% (median 3 *vs*. 2, *P* < 0.001) compared with the TSILC group. In the high BMI subgroup (≥ 24 kg/m^2^), the operation time was decreased by 17.5% (83.43 ± 17.24 *vs*. 101.13 ± 18.12 minutes, *P* = 0.012), and the gallbladder triangle separation time was reduced by 50% (median 1 *vs*. 2 minutes, *P* = 0.029) for MSILC. No cases in eith er group required conversion to laparotomy or experienced severe complications. No significant differences were observed in the 24-hour postoperative pain score (median visual analog scale score 2 *vs*. 2, *P* = 0.982) and cosmetic satisfaction regarding the incision 1 month after surgery (median 5 *vs*. 4, *P* = 0.221) between the MSILC and TSILC groups. However, consumables cost and total hospitalization costs in the MSILC group increased by 34.7% (*P* < 0.001) and 14.4% (*P* = 0.042), respectively.

**Conclusion:**

MSILC significantly improves intraoperative efficiency by optimizing the trocar layout, especially in patients with high BMI, while maintaining safety and cosmetic outcomes comparable with those of TSILC. However, cost increases due to the use of microdevices should be taken into consideration.

## Introduction

Gallbladder disease has a high global incidence, with approximately 10–20% of adults affected by cholelithiasis each year. In the United States, approximately 6% of men and 9% of women have cholelithiasis (Everhart et al., 1999), necessitating more than 700,000 cholecystectomies annually (Shaffer, 2006). The incidence of cholelithiasis in China is 9–10% (Zhang et al., 2015). Conventional laparoscopic cholecystectomy, first performed by Mouret in 1987 (Mouret, 1996), with four incisions, is gradually developing into the gold standard for the treatment of benign gallbladder diseases (Yamashita et al., 2007). Despite their efficacy, concerns regarding scarring caused by multiple incisions and postoperative chronic pain have prompted continuous innovations in minimally invasive techniques (Alhashemi et al., 2017). In minilaparoscopic cholecystectomy, 3–5 mm micro-instruments are used to reduce the total length of the incision, improving the appearance of postoperative scars and reducing postoperative pain (De Carvalho, Fierens & Kint, 2013). However, this approach reduces the maneuverability of instruments and limits the surgical field of view (Nam et al., 2024). In patients with a high body mass index (BMI), limited operating space increases the technical difficulty of the procedure (Kim & Jang, 2019). However, some patients have higher cosmetic expectations, seeking further reduction of surgical incision-associated scars. Navarra et al. (1997) performed the first single-incision laparoscopic cholecystectomy (SILC), which achieves a scarless appearance through a single umbilical incision; however, its coaxial instrument operation leads to a restricted surgical field of view, frequent instrument collisions, and steep learning curves, limiting its clinical applicability (Rawlings et al., 2010). In patients with obesity, TSILC further increases surgery-associated risks owing to the narrow abdominal cavity space and requires prolonged operation times (Jang et al., 2014).

To address these limitations, this study proposed minilap-assisted modified SILC (MSILC), in which the trocar layout is optimized by deploying two adjacent trocars at the umbilicus, combined with a 2-mm auxiliary hole created beneath the right costal margin, while retaining the cosmetic advantages of TSILC. Although termed ‘Modified Single-Incision’, the MSILC technique utilizes a clustered multi-trocar configuration at the umbilicus, combined with a 2-mm auxiliary subcostal port, to mitigate the intrinsic limitations of pure TSILC while preserving its cosmetic intent. Additionally, the use of micro-instruments improves the triangular operating space and reduces instrument conflicts to achieve or approach the advantages of minilaparoscopic cholecystectomy. This study retrospectively compared MSILC with TSILC, focusing on operator convenience (the number of intraoperative instrument conflicts, Surgical difficulty score, and operator comfort), operation time, and clinical outcomes, especially in the high BMI subgroup (BMI ≥ 24 kg/m^2^). The aim was to explore a new procedure that was minimally invasive, operationally efficient, and safe to provide evidence-based guidance for personalized surgical decision-making.

## Methods

This study retrospectively enrolled patients diagnosed with gallbladder polyps and/or gallbladder stones who underwent MSILC or TSILC at the Department of Hepatobiliary and Pancreatic Surgery at the eighth affiliated hospital of Sun Yat-sen University between December 2021 and April 2025.

The inclusion criteria were as follows: (1) two or more preoperative imaging diagnoses of gallbladder stones with chronic cholecystitis, gallbladder polyps, gallbladder adenomyosis, and other benign gallbladder diseases, (2) surgical management with MSILC or TSILC. The exclusion criteria were as follows: (1) previous history of abdominal surgery, (2) postoperative pathological confirmation of gallbladder malignancy, (3) conversion to multiport (*e.g.*, conventional four-port) laparoscopic cholecystectomy or to open surgery during the procedure, and (4) presence of concomitant umbilical or paraumbilical hernia requiring repair, (5) incomplete follow-up data. The primary observational endpoint was the number of intraoperative instrument conflicts, and the secondary observational endpoints were operation time, cosmetic satisfaction regarding the incision scar, operator comfort, and Surgical difficulty score.

All patients were exempted from informed consent. All surgeries were performed by the same surgeon, who had performed more than 100 LCs. The study was reviewed and approved by the institutional Ethics Committee (Ethics Research Ethics of the Eighth Affiliated Hospital of Sun Yat-sen University 2025-078-01).

### Surgical procedures

Single-port laparoscopic cholecystectomy (SILC) involves making a 2.5-cm transverse incision within the infra-umbilical fold, dissecting into the abdomen layer-by-layer, inserting a disposable single-port operation kit, inserting a 30° mirror and operating instruments. The subsequent critical steps—including dissection of the hepatocystic triangle, management of the cystic duct and artery (adhering to the same clipping protocol), detachment of the gallbladder from the liver bed, hemostasis check, and specimen retrieval—were performed consistently with the standardized MSILC procedure as detailed in the previous section.

Minilap-assisted modified single-port laparoscopic cholecystectomy (MSILC) was performed in accordance with the standard operating procedure (SOP) detailed below.

(1) Trocar layout and puncture positioning

Umbilical double channel: Make a 10-mm arc incision below the umbilicus which the 30° laparoscope was introduced, and insert one 5-mm trocars at three points around the umbilicus as the primary operating channel. The spacing should be carefully maintained at 2–5 mm to ensure space for instrument maneuverability and reduce interference.

**Figure 1:**
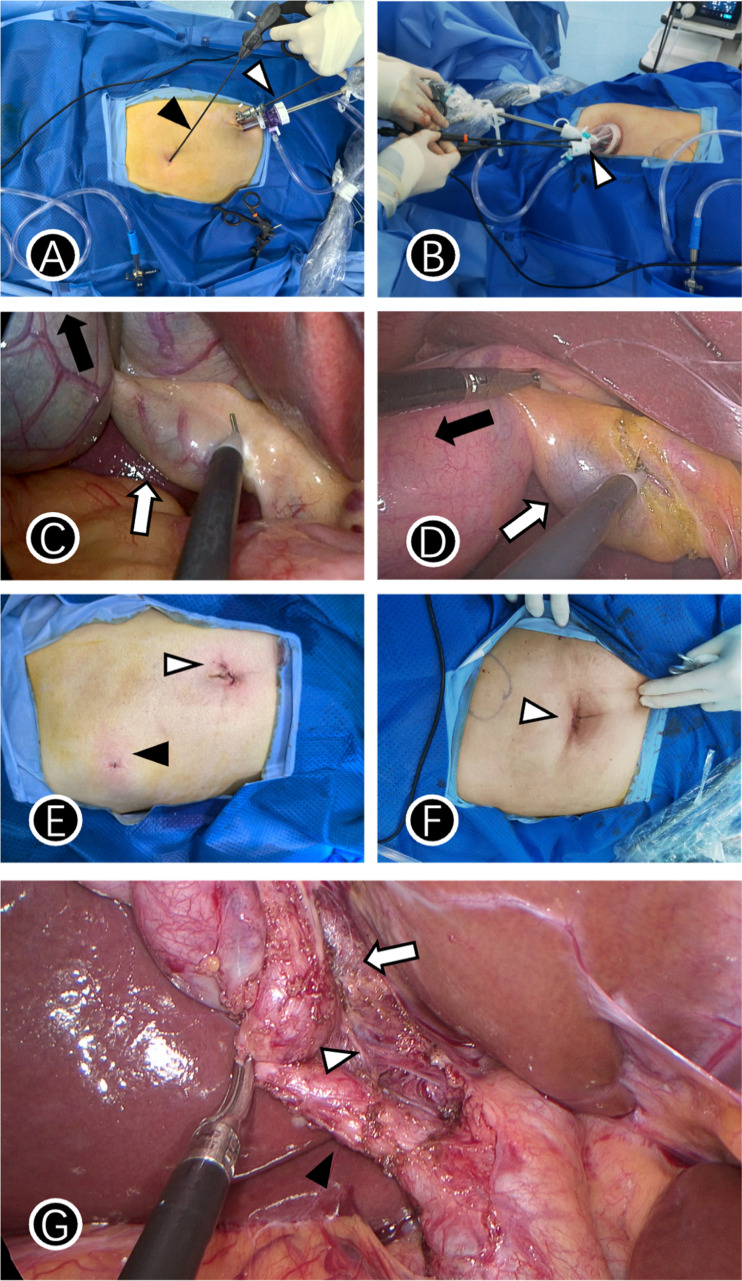
Trocar layout and postoperative wounds in two groups. (A) The black triangle indicates the 2-mm minilap and the white triangle indicates the umbilical observation hole and the main operation hole in MSILC group; (B) white triangle indicates a single-port access device in a SILC group; (C) black arrow indicates the pulling direction of the 2-mm minilap and the white arrow indicates the direction of the main operation hole in MSILC group; (D) black arrow indicates the pulling direction of the secondary operation hole, and the white arrow indicates the direction of the main operation hole of a Single-port access device in SILC group; (E) black arrow indicates the 2-mm minilap incision, and the white arrow indicates the periumbilical incision in the MSILC group; (F) white arrow indicates the periumbilical incision in the SILC group; (G) CVS was clearly exposed in MSILC group, the black triangle indicates cystic duct, the white triangle indicates cystic artery, white arrow indicates the lower one-third of the gallbladder bed is dissected.

Auxiliary hole beneath the right costal margin: Insert a 2-mm minilap discreetly at the junction of the right midclavicular line and costal arch (Fig. 1A).

(2) Instrument configuration and establishment of the surgical field:

Use a 30° high-definition laparoscope to provide a wide-angle field of view, and insert all instruments into the abdominal cavity through a preset channel.

Maintain the pneumoperitoneum pressure at 12–14 mmHg. Dynamically adjust the patient’s position during the operation to a 30° head-up and left-tilted orientation for optimal exposure of the gallbladder triangle.

(3) Key operation module

Gallbladder triangle exposure: Adopt the four-step method as follows. (1) The gallbladder neck was retracted superiorly and to the right using a grasper introduced through the 2-mm subcostal auxiliary port, (2) a right-angle forceps was inserted through the umbilical 5-mm main operating port to bluntly dissect the anterior sheath of the cystic duct (Fig. 1C) and the tissues within the hepatocystic triangle until the Critical View of Safety (CVS) was clearly exposed—that is, only the cystic duct and the cystic artery were confirmed to be entering the gallbladder (Fig. 1G). (3) After the CVS was unequivocally achieved, the cystic artery and cystic duct were individually clipped and transected. After the Critical View of Safety was unequivocally achieved, the cystic duct and cystic artery were secured and transected following a standardized protocol. For the cystic duct, two Hem-o-lok clips were applied on the proximal side (towards the common bile duct) and one clip on the distal side (towards the gallbladder) before transection between them. For the cystic artery, one Hem-o-lok clip was applied on the proximal side, and the distal side was sealed using electrocautery before transection.

Gallbladder dissection and removal: following dissection of the gallbladder along the liver bed, a sterile retrieval bag was introduced through the 10-mm umbilical port to avoid bile contamination and facilitate specimen removal. This 10-mm port below the umbilicus was also used for specimen retrieval upon completion of the dissection. The port site was closed using a standard layered technique. The fascial layer was reapproximated with absorbable sutures to ensure a secure closure and prevent postoperative port-site hernia. The skin was then closed with a subcuticular suture for optimal cosmetic results.

(4) Risk management of high-risk cases

For patients with BMI ≥ 24 kg/m^2^ or gallbladder wall thickness ≥ three mm, additionally use intraoperative laparoscopic ultrasound scanning to confirm anatomical variations of the cystic duct; in case of severe adhesions, pre-place Hem-o-lok clamps at the proximal end of the cystic duct to reduce the risk of bile leakage and bleeding.

Single-port laparoscopic cholecystectomy (SILC) involves making a 2.5-cm transverse incision within the infra-umbilical fold, dissecting into the abdomen layer-by-layer, inserting a disposable single-port operation kit, inserting a 30° mirror and operating instruments (Fig. 1B). The subsequent critical steps—including dissection of the hepatocystic triangle, management of the cystic duct and artery (Fig. 1D) (adhering to the same clipping protocol), detachment of the gallbladder from the liver bed, hemostasis check, and specimen retrieval—were performed consistently with the standardized MSILC procedure as detailed in the previous section.

### Observation indicator definition

The number of intraoperative instrument conflicts refers to the total number of instrument collisions, visual field obstructions, or stagnations during the operation, which was recorded in real time by the first assistant within 10 min after the operation.

The surgical difficulty scoring system assessed and quantified using the Parkland grading scale (Madni et al., 2018), was used to evaluate the difficulty of dissection during cholecystectomy, and comprised the following five items (0–3 points for each item, resulting in 0–15 points for the total score; a higher score indicated greater difficulty): (1) Fat coverage: 0 = no fat, 1 = mild, 2 = moderate, 3 = severe; (2) adhesion degree: 0 = no adhesion, 1 = membranous, 2 = dense fibrous, 3 = scarring; (3) time spent dissecting the gallbladder triangle: 0 = <10 min, 1 = 10–20 minutes, 2 = 20–30 minutes, 3 = >30 min; (4) intraoperative bleeding: 0 = no bleeding, 1 = a small amount of bleeding, 2 = electrocoagulation is required for hemostasis, 3 = conversion to laparotomy is required; (5) difficulty of surgical field exposure: 1 = excellent exposure, 2 = partial obstruction, 3 = frequent adjustment is required, 4 = severe difficulty, 5 = exposure is unfeasible and conversion to laparotomy is required. Scoring was performed independently by the surgeon and the first assistant within 10 min after the operation. If the difference in sub-item scores was ≥2 points, the final score was determined by an independent senior surgeon after blind review of the video.

Operator comfort was quantitatively evaluated using a modified Likert 5-point scale: 1 point (extremely uncomfortable) indicated frequent instrument conflicts, difficulty in achieving surgical field exposure, and requirement of repeated adjustments of body position or instruments and multiple pauses to relieve operator fatigue; 2 points (uncomfortable) indicated frequent instrument conflicts, requirement of additional traction instruments for surgical field exposure, and feasibility of operation completion despite limited operation fluency; 3 points (average) indicated general instrument conflicts, satisfactory surgical field exposure, and no obvious interruptions during the operation; 4 points (comfortable) indicated fewer instrument conflicts, good surgical field exposure, and smooth operation without the requirement of additional adjustments; 5 points (extremely comfortable) indicated few instrument conflicts, ideal surgical field exposure, instrument operation in line with ergonomic design, and no fatigue throughout the process. The surgeon independently completed scoring within 10 min after the end of the operation.

### Visual analog scale (VAS) score 24 h after surgery

The pain intensity 24 h after surgery was evaluated using a 10-cm linear VAS scale with “no pain” (0 points) and “unbearable severe pain” (10 points) marked at both ends. At 24 h postoperatively, patients marked their subjective pain level on the scale, and an independent observer measured the distance from the marked point to the “no pain” end of the scale (accurate to millimeters) and converted it into a 0–10 score.

Cosmetic satisfaction regarding the incision scar was evaluated by patients at the 1-month follow-up: (1) very dissatisfied/very poor—extremely negative experience, far below expectations with significant issues; (2) unsatisfied/poor—negative experience with most aspects not meeting expectations and requiring improvements; (3) average—acceptable neutral experience, with partial satisfaction of expectations without notable benefits; (4) satisfied/good—positive experience, mostly met or slightly exceeded expectations without obvious defects; (5) very satisfied/very good—excellent experience, far exceeded expectations with excellent performances in all aspects.

### Sample size calculation

This study used a non-parametric sample size calculation formula based on the median difference in the number of intraoperative instrument conflicts. According to the pre-test data (*n* = 10 per group), the median difference in intraoperative instrument conflicts between the MSILC and TSILC groups was 10 (15 *vs.* 25), and the combined interquartile range (IQR) was 15. The effect size (Cliff’s delta, *δ*) was calculated as follows:

\begin{eqnarray*}\delta = \frac{2 \left( \mathrm{U}- \frac{{\mathrm{n}}_{1}{\mathrm{n}}_{2}}{2} \right) }{{\mathrm{n}}_{1}{\mathrm{n}}_{2}} = \frac{2 \left( 175-50 \right) }{100} =0.5 (\text{Pilot data}:\mathrm{U}=175,{\mathrm{n}}_{1}={\mathrm{n}}_{2}=10) \end{eqnarray*}

where UU is the Mann–Whitney U statistic. The significance level and statistical power were set at α = 0.05 (two-sided) and 1−β = 0.8, respectively, and the sample size was calculated using the Noether formula:

\begin{eqnarray*}{n}_{\text{per group}}= \frac{{ \left( {\mathrm{Z}}_{1-\mathrm{\alpha }/2}+{\mathrm{Z}}_{1-\mathrm{\beta }} \right) }^{2}}{3{\delta }^{2}} = \frac{{ \left( 1.96+0.84 \right) }^{2}}{3\times 0.{5}^{2}} \approx 21\mathrm{subjects} \end{eqnarray*}

Considering a dropout rate of 10%, 25 cases were required in each group, with a total sample size of 50 cases and an actual statistical power of 82%.

### Statistical analysis

The normality of the distributions of continuous variables was evaluated using the Shapiro–Wilk normality test. Normally distributed variables are expressed as mean ± standard deviation, and the independent sample *t*-tests were used for intergroup comparison. Non-normally distributed variables are expressed as median (interquartile range), and Mann–Whitney U tests were used for intergroup comparison. Categorical variables are described as frequencies (percentages), and differences between groups were analyzed using the chi-square test or Fisher’s exact test, as appropriate. The primary endpoint (number of intraoperative instrument conflicts) and secondary endpoints (operative time and surgical difficulty score) were compared between groups using Mann–Whitney U tests, and the effect size was calculated using Cliff’s delta (*δ*), where *δ* > 0.33 indicated clinically significant differences. Subgroup analysis was performed based on BMI stratification (≥24 kg/m^2^ *vs.* < 24 kg/m^2^) using stratified nonparametric tests, and the interactions were evaluated using generalized linear models. Bonferroni correction was used for multiple comparisons (pre-set six endpoints, α = 0.008 after correction). All analyses followed the intention-to-treat principle, with a significance level of α = 0.05 (two-sided). A *P*-value < 0.05 was considered statistically significant. Data analysis was performed using SPSS^®^ version 29.0 (IBM Corporation, USA), and graphs were generated using GraphPad Prism^®^ 9.4.0.

## Results

A total of 51 patients who underwent cholecystectomy were included: 25 in the MSILC and 26 in TSILC groups. No statistically significant differences were observed in baseline data, including age, BMI, gallbladder wall thickness, or American Society of Anesthesiologists grade between the two groups (*P* > 0.05). The proportion of men was significantly higher in the MSILC group than in the TSILC group (60% *vs.* 15.4%, *P* = 0.001), which may have been due to sample selection bias; however, sex-stratified analysis showed no significant effect of sex on surgical outcomes (Table 1).

**Table 1:** Preoperative baseline characteristics of the patients in two group.

Baseline characteristics	MSILC (*n* = 25)	TSILC (*n* = 26)	*p* value
Age (yesr)	44.48 ± 12.34	39.85 ± 8.89	0.129
Sex (Male)	15 (60)	4 (15.4)	0.001
BMI (kg/m^2^)	24.11 ± 3.16	23.67 ± 4.04	0.666
Gallbladder wall thickness	2.44 (1.14, 9.64)	2.79 (1.28, 6.32)	0.843
Gallbladder disease (Gallstones)	20 (80)	20 (76.9)	0.530
ASA classification (Level I–III)	24(96)	25(96.2)	0.745

The number of instrument conflicts was significantly lower in the MSILC group than in the TSILC group (median: 16 *vs.* 26, *P* < 0.001). No significant difference was observed in the total operation time between the MSILC and TSILC groups (88.52 ± 20.81 min *vs.* 95.35 ± 19.03 min, *P* = 0.227). The Surgical difficulty score (median 8 *vs.* 11.5 points, *P* = 0.007) and the difficulty score for surgical field exposure (median 2 *vs.* 3.5 points, *P* = 0.006) were lower for MSILC than for TSILC. The degree of adhesion (*P* = 0.004) and the time required for gallbladder triangle separation (*P* = 0.015) were better in the MSILC group than in the TSILC group. Operator comfort was significantly better in the MSILC group compared with the TSILC group (median 3 *vs.* 2, *P* < 0.001). No significant difference was observed in the amount of blood loss between the two groups (10 *vs.* 10 mL, *P* = 0.506) (Table 2). No cases in either group required conversion to traditional three-port or four-port laparoscopic cholecystectomy or laparotomy during surgery.

**Table 2:** Intraoperative characteristics of the patients in two group.

Variables	MSILC (*n* = 25)	TSILC (*n* = 26)	*p* value
Operation time (minutes)	88.52 ± 20.81	95.35 ± 19.03	0.227
Numbe of intraoperative instrument _conflicts	16 (7, 22)	26 (22, 30)	<0.001
Operator comfort	3 (1, 3)	2 (1, 3)	<0.001
Nassar Rating	8 (1, 15)	11.5 (5, 15)	0.007
-Fat coverage	2 (0, 3)	2 (1, 3)	0.101
-Degree of adhesion	1 (0, 3)	2 (1, 3)	0.004
-Required_for_dissection	1 (0, 3)	2 (1, 3)	0.015
-Intraoperative_blood_loss	1 (0, 2)	1.5 (0, 2)	0.089
-Difficulty of surgical field exposure	2 (1, 4)	3.5 (2, 4)	0.006
Intraoperative blood_loss	10 (5, 50)	10 (2, 50)	0.506
Conversion to laparotomy rate	0	0	–

No significant differences were observed in the VAS pain score 24 h after surgery (median 2 points *vs.* 2 points, *P* = 0.982), incidence of complications within 30 days (0% *vs.* 0%), and the length of hospital stay (median 7 days) between the two groups (Table 3). At the 1-month follow-up, no significant difference was observed in the cosmetic score regarding the incision scars between the two groups (median 5 points *vs.* 4 points, *P* = 0.221). In MSILC, the 2-mm auxiliary hole was located in the hidden part of the right costal margin, leading to a small scar (Fig. 1E); the umbilical incision scars in the MSILC group were similar to those in the TSILC group (Fig. 1F).

**Table 3:** Early outcomes of the patients in two group.

Variables	MSILC (*n* = 25)	TSILC (*n* = 26)	*p* value
VAS _score _24 _hours _after _surgery	2(1, 3)	2 (2, 3)	0.982
Complications _within _30 _days_ after_ surgery	0	0	–
Length _of _hospital _stay(Days)	7 (4, 16)	7 (4, 19)	0.592
Wound _cosmetic _satisfaction	5 (3, 5)	4 (2, 5)	0.221

The common inclusions of anesthesia costs are rre-anesthesia evaluation, anesthesia procedures, intra-anesthesia management and post-anesthesia recovery. Anesthesia consumable fees is a composite item, whose value equals the sum of “anesthesia fees” and the “surgical consumable fees” incurred during the operation. The cost of consumables was 34.7% higher in the MSILC group than in the TSILC group (10,528.12 ± 2,437.37 yuan *vs.* 7,818.80 ± 2,131.61 yuan, *P* < 0.001), mainly due to the use of micro-devices. The total hospitalization cost was significantly higher in the MSILC group than in the TSILC group (22,014.65 ± 3,936.83 yuan *vs.* 19,242.72 ± 5,381.60 yuan, *P* = 0.042) (Table 4).

**Table 4:** Hospitalization_expenses of the patients in two group.

Variables	MSILC (*n* = 25)	TSILC (*n* = 26)	*p* value
Total hospitalization_expenses (yuan)	22,014.65 ± 3936.83	19,242.72 ± 5381.60	0.042
Consumables_cost (yuan)	10,528.12 ± 2437.37	7818.80 ± 2131.61	<0.001
Anesthesia_costs (yuan)	2,687.34 ± 391.44	2763.17 ± 367.05	0.479
Consumables_anesthesia _costs (yuan)	12678.47 ± 2028.40	10,695.55 ± 2343.36	0.001

**Figure 2:**
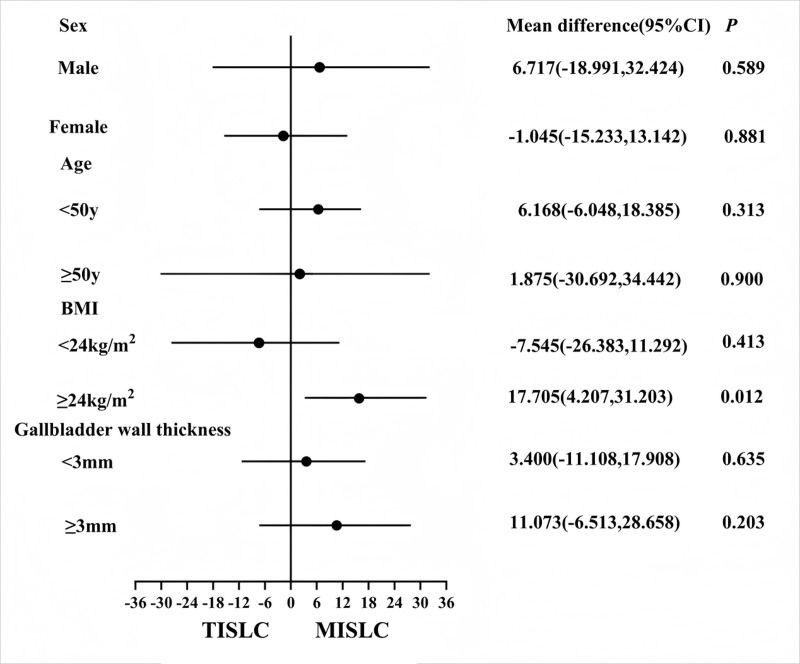
Forest plot of surgical time for patients with different combinations of instruments in different subgroups.

In the high BMI subgroup (BMI ≥24 kg/m^2^), the operation time was significantly decreased for MSILC (83.43 ± 17.24 min *vs.* 101.13 ± 18.12 min, *P* = 0.012) (Table 5). In the normal BMI subgroup (BMI < 24 kg/m^2^), no difference was observed in the operation time between the two procedures (95.00 ± 23.87 min *vs.* 87.45 ± 18.09 min, *P* = 0.413) (Table 6). Table 7 presents the results of the subgroup analysis of operation time stratified by sex, age, BMI, and gallbladder wall thickness. The analysis revealed that there were no statistically significant differences in operation time between MSILC and TSILC in the subgroups of sex, age, or gallbladder wall thickness (all *P* > 0.05). However, in the high-BMI subgroup (BMI ≥ 24 kg/m^2^), the operation time was significantly shorter for MSILC than for TSILC (83.43 ± 17.24 *vs.* 101.13 ± 18.12 min, *P* = 0.012). The forest plot (Fig. 2) further showed that the advantages of MSILC for patients with high BMI were consistent (95% CI [4.207–31.203]).

**Table 5:** High BMI subgroup analysis of intraoperative characteristics of the patients in two group.

Variables	MSILC (*n* = 14)	TSILC (*n* = 15)	*p* value
Operation time(minutes)	83.43 ± 17.24	101.13 ± 18.12	0.012
Numbe of intraoperative instrument_conflicts	16.5 (7, 22)	27 (24, 30)	<0.001
Operator comfort	3.5 (1, 5)	1 (1, 3)	0.002
Nassar Rating	8 (2, 15)	15 (9, 15)	0.016
-Fat coverage	2 (0, 3)	3 (2, 3)	0.134
-Degree of adhesion	1.5 (0, 3)	3 (1, 3)	0.029
-Required_for_dissection	1.5 (0, 3)	3 (2, 3)	0.029
-Intraoperative_blood_loss	1 (0, 2)	2 (1, 2)	0.134
-Difficulty of surgical field exposure	2.5 (1, 4)	4 (3, 4)	0.029
Intraoperative blood_loss	10 (5, 50)	10 (5, 50)	0.186
Conversion to laparotomy rate	0	0	–

**Table 6:** Normal BMI subgroup analysis of intraoperative characteristics of the patients in two group.

Variables	MSILC (*n* = 11)	TSILC (*n* = 11)	*p* value
Operation time(minutes)	95 ± 23.87	87.45 ± 18.09	0.413
Numbe of intraoperative instrument_conflicts	15 (12, 19)	25 (22, 28)	<0.001
Operator comfort	3 (2, 5)	2 (1, 3)	0.005
Nassar Rating	6.91 ± 4.81	9.36 ± 3.33	0.179
-Fat coverage	1 (0, 3)	2 (1, 3)	0.519
-Degree of adhesion	1 (0, 3)	2 (1, 3)	0.076
-Required_for_dissection	1 (0, 3)	3 (2, 3)	0.332
-Intraoperative_blood_loss	1 (0, 2)	2 (0, 2)	0.438
-Difficulty of surgical field exposure	2 (1, 4)	3 (3, 4)	0.151
Intraoperative blood_loss	10 (5, 20)	10 (2, 50)	0.562
Conversion to laparotomy rate	0	0	–

**Table 7:** Comparison of surgical time in different subgroups of patients in two groups.

Variables	MSILC	TSILC	Difference value (95% CI)	*t* value	*p* value
Sex					
Male	82.53 ± 23.20	89.25 ± 12.07	6.717 (−18.991, 32.424)	0.551	0.589
Female	97.50 ± 12.96	96.45 ± 20.05	−1.045 (−15.233, 13.142)	−0.150	0.881
Age (years)					
<50	91.06 ± 19.20	97.23 ± 18.26	6.168 (−6.048, 18.385)	1.023	0.313
≥50	83.13 ± 24.34	85.00 ± 22.73	1.875 (−30.692, 34.442)	0.128	0.900
BMI (kg/m^2^)					
<24	95.00 ± 23.87	87.45 ± 18.09	−7.545 (−26.383, 11.292)	−0.836	0.413
≥24	83.43 ± 17.24	101.13 ± 18.12	17.705 (4.207, 31.203)	2.691	0.012
Gallbladder_wall_thickness (mm)					
<3	85.40 ± 22.06	88.80 ± 16.31	3.400 (−11.108, 17.908)	0.480	0.635
≥3	93.20 ± 18.89	104.27 ± 19.53	11.073 (−6.513, 28.658)	1.318	0.203

## Discussion

In recent years, advancements in surgical instruments and technologies for minimally invasive laparoscopy have further reduced postoperative pain and scarring (Raman, Bagrodia & Cadeddu, 2009). Compared with the traditional four-port laparoscopic cholecystectomy, patients prefer single-incision or less traumatic laparoscopic surgery (Bucher, Pugin & Morel, 2009). However, compared with conventional laparoscopic surgery, SILC has certain limitations: (1) in Traditional single-incision laparoscopic cholecystectomy (SILC) typically requires a larger umbilical incision. A meta-analysis including 927 patients indicated that the incidence of port-site hernias (specifically umbilical hernias) was significantly higher in SILC than in conventional multiport laparoscopic cholecystectomy (4.0% *vs.* 1.1%, OR 2.50) (Haueter et al., 2017); (2) due to the reduced range of the instrument in the abdominal cavity, the “chopstick effect” leads to the “hand collision” issue (Rawlings et al., 2010); (3) the inconsistent positioning of the camera and the operating instrument leads to a reduced surgical field of view (Haber et al., 2010); and (4) because all instruments are concentrated on the umbilicus, traditional single-incision laparoscopic cholecystectomy (SILC) typically requires a larger umbilical incision. A meta-analysis including 927 patients indicated that the incidence of port-site hernias (specifically umbilical hernias) was significantly higher in SILC than in conventional multiport laparoscopic cholecystectomy (4.0% *vs.* 1.1%, OR 2.50) (Haueter et al., 2017).

This study proposes the novel technique MSILC, whose core innovation includes the use of a 2-mm micro-assisted port under the right costal margin, combined with a trocar layout of two adjacent channels at the umbilicus. Standardizing the surgical procedure while retaining the cosmetic advantages of single-port laparoscopy significantly improves the operative efficiency and reduces procedural difficulty. Herein, in the MSILC group, the number of instrument conflicts was reduced by 38.5% (median 16 *vs.* 26, *P* < 0.001), the operator’s comfort score was increased by 50% (median 3 *vs.* 2, *P* < 0.001), and the Surgical difficulty score was decreased by 30.4% (median 8 *vs.* 11.5, *P* = 0.007) compared with the TSILC group (Table 2). These results are consistent with the findings of Dabbagh et al. (2015), which revealed that micro-laparoscopy could shorten the operation time due to a clearer surgical field, thereby improving operation efficiency. Herein, in the high BMI subgroup (BMI ≥ 24 kg/m^2^), the operation time was 17.5% shorter (83.43 ± 17.24 *vs.* 101.13 ± 18.12 min, *P* = 0.012) and the time for dissecting the gallbladder triangle was significantly reduced (median 1 *vs.* 2, *P* = 0.029) with MSILC compared with TSILC (Table 5). This suggests that the microauxiliary hole effectively alleviates the spatial limitation of the single-port technique in coaxial operation, and a distance of 2–5 mm from the umbilicus can effectively reduce the collision of instruments between the observation hole and the main operation hole, especially in patients with obesity, by increasing the freedom of movement of the instrument and improving the exposure of the gallbladder triangle, which is consistent with the study of Hussien et al. (2002), which can significantly improve the quality of triangular exposure in patients with obesity.

Our analyses revealed no statistical difference in the aesthetic score for incision-related scars between the two groups (median, 5 *vs.* 4; *P* = 0.221). The auxiliary hole used in MSILC measured only 2-mm in diameter and was positioned discreetly in the rib margin, achieving a “visually seamless” effect similar to traditional single-port laparoscopic surgery (Fig. 1E). This is consistent with the findings of Lee et al. (2010) regarding the aesthetic advantages of single-port surgery. However, the consumables cost of MSILC increased by 34.7% compared with TSILC (10,528.12 ± 2,437.37 *vs.* 7,818.80 ± 2,131.61 yuan, *P* < 0.001), mainly due to the cost of specialized micro-instruments used. This contradiction highlights a classic dilemma in the development of minimally invasive technology: the balance between technological innovation and medical resource allocation. A randomized controlled study by Bucher et al. (2011) indicated that the cost of single-port technology was higher than that of traditional laparoscopic cholecystectomy and that the increase in cost was mainly due to the cost of the consumables used during single-port surgery. In the future, a cost-utility model should be established to quantify its economic value and balance the operator’s experience, patient satisfaction, medical costs, and the actual condition of the patient to guide better clinical decision-making (Li et al., 2023).

This study showed that MSILC did not increase the risk of postoperative complications; no statistically significant difference was observed in pain scores (median VAS score 2 *vs.* 2), complication rates (0% *vs.* 0%), and hospital stay (median 7 days) between the two groups (Table 3). A randomized controlled trial has shown that SILC offers comparable surgical safety, intraoperative blood loss, hospital stay, and postoperative complication rates to those of traditional laparoscopic surgery. A meta-analysis showed that SILC had certain advantages over standard laparoscopic surgery, such as improved aesthetic outcomes and higher patient satisfaction regarding the incisions and less postoperative pain (Hussien et al., 2002); however, the operation time was slightly longer, and the incidence of conversion to conventional laparoscopic surgery was higher (Navarra et al., 1997). However, Marks et al.’s (2013) RCT study showed that there was no significant difference between single-port laparoscopic cholecystectomy and traditional laparoscopic surgery in terms of surgical safety, surgical blood loss, hospitalization period, and postoperative complication rate compared with the control group. In our study, no cases required conversion to conventional laparoscopic cholecystectomy, which may be related to the small sample size. Notably, the MSILC group had a significantly better operation time for the gallbladder triangle than the TSILC group, suggesting that although no significant difference in surgical complications was observed between the two groups, the auxiliary hole may improve the stability of the surgical field exposure, thereby improving surgical safety. In this study, the improved operating comfort provided by MSILC enhances the reproducibility of the standardized surgical process, improving surgical safety and reducing postoperative complications.

This study established a standardized operating procedure for MSILC to balance technical complexity and operational reproducibility. Based on the concept of “scarless” surgery, the trocar layout should be strictly standardized: the distance between the two channels at the umbilicus is recommended to be 2–5 mm to ensure the space for instrument maneuverability, and the two mm auxiliary hole beneath the right costal margin should be located at the intersection of the midclavicular line and the costal arch. The safety of the procedure conforms to the single-port surgery consensus proposed by Gill et al. (2010); the instrument selection adopts the principle of “minimally invasive first”, and the key steps of the surgery using 30° high-definition laparoscopy combined with 5-mm trocars follow the “modular” operation. The graded management strategy of the Tokyo Guidelines should be referred to; additionally, the use of intraoperative ultrasound guidance or pre-placement of hemostatic clips is recommended (Yamashita et al., 2007). The standard operating procedure described herein will help form a consensus regarding surgical techniques that are both technically feasible and resource-accessible in the future and provide systematic evidence supporting the widespread clinical application of MSILC.

This study has three limitations. First, the single-center retrospective design may have resulted in selection bias, especially in the high BMI subgroup; additionally, the statistical power was limited, and the quality control standards for retrospective studies should be referred to for improvement. Second, data regarding scar assessment and port-site hernia incidence more than 6 months after surgery were lacking . Third, assessments of instrument collisions and surgical difficulty herein were subjective evaluations of the surgeon and the first assistant, as no objective evaluation standard currently exists. This study achieved aesthetic outcomes similar to those of TSILC while improving the surgical efficiency and reducing procedural difficulty, especially shortening the operation time in patients with high BMI. However, in the future, higher-level evidence is required to corroborate the effectiveness, feasibility, and safety of this surgical method.

## Conclusion

MSILC significantly reduces intraoperative instrument conflicts, lowers surgical difficulty, and improves the surgeon’s comfort by optimizing the layout of trocars. It can especially shorten the operation time for patients with high BMI, and is comparable to TSILC in terms of safety and cosmetic results. However, it should be noted that the cost of its consumables is higher. In clinical application, it is necessary to balance the technical advantages and medical costs to provide patients with personalized surgical options.

## Supplemental Information

10.7717/peerj.20807/supp-1 Supplemental Information 1 Data

## Additional Information and Declarations

### Data Availability

The following information was supplied regarding data availability: The raw data is available in the Supplemental Files.
