# Supplementary figures and images for: Minilap-assisted modified single-incision vs. traditional single-incision laparoscopic cholecystectomy: a retrospective cohort study on instrument conflict and operative efficiency
Source: PeerJ. 2026 Mar 10;14:e20807. doi: 10.7717/peerj.20807 (PMC12985008; doi:10.7717/peerj.20807)

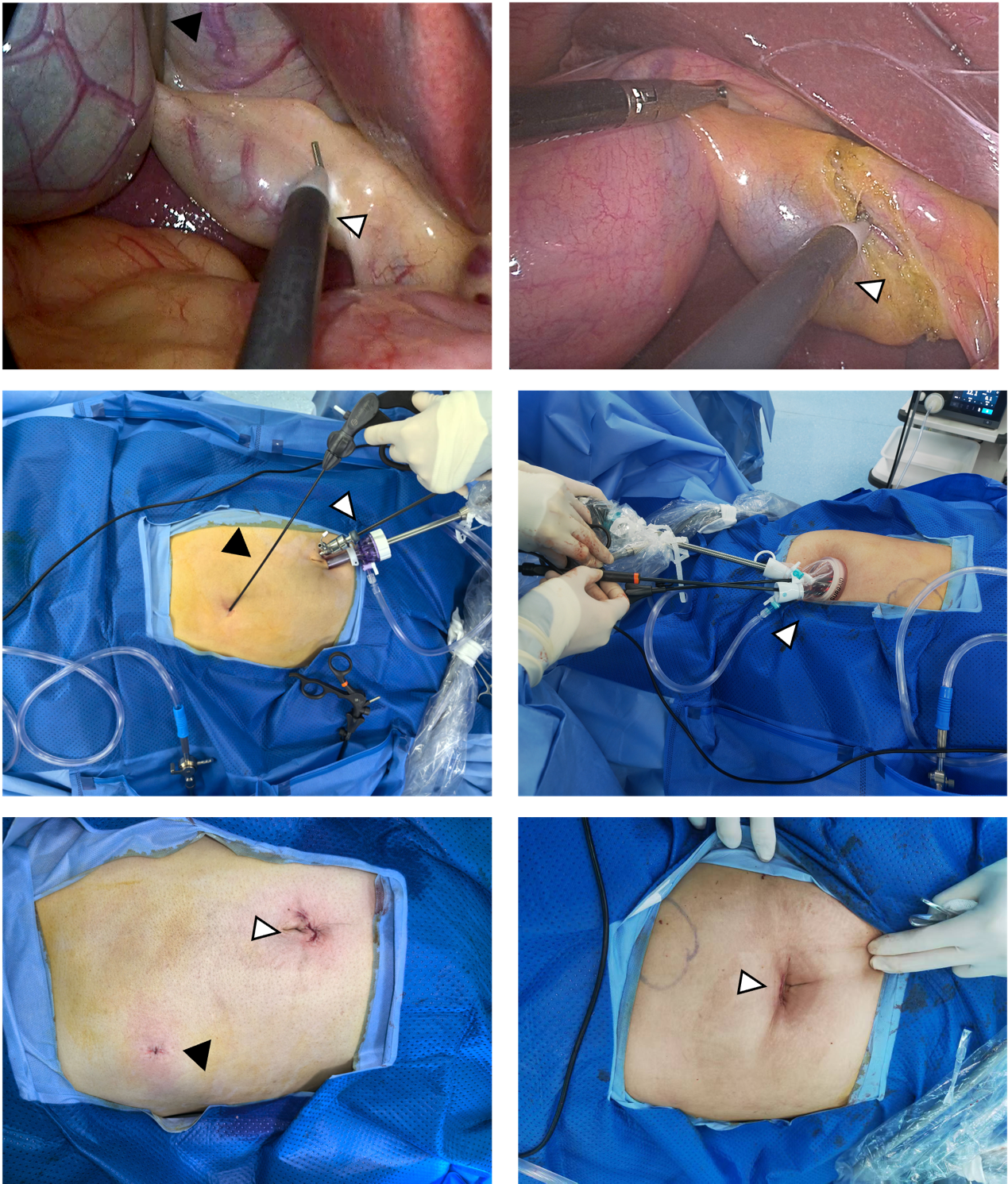

Supplement: Supplemental Information 1 [file peerj-14-20807-s001.zip › Figure1/Figure1.png]

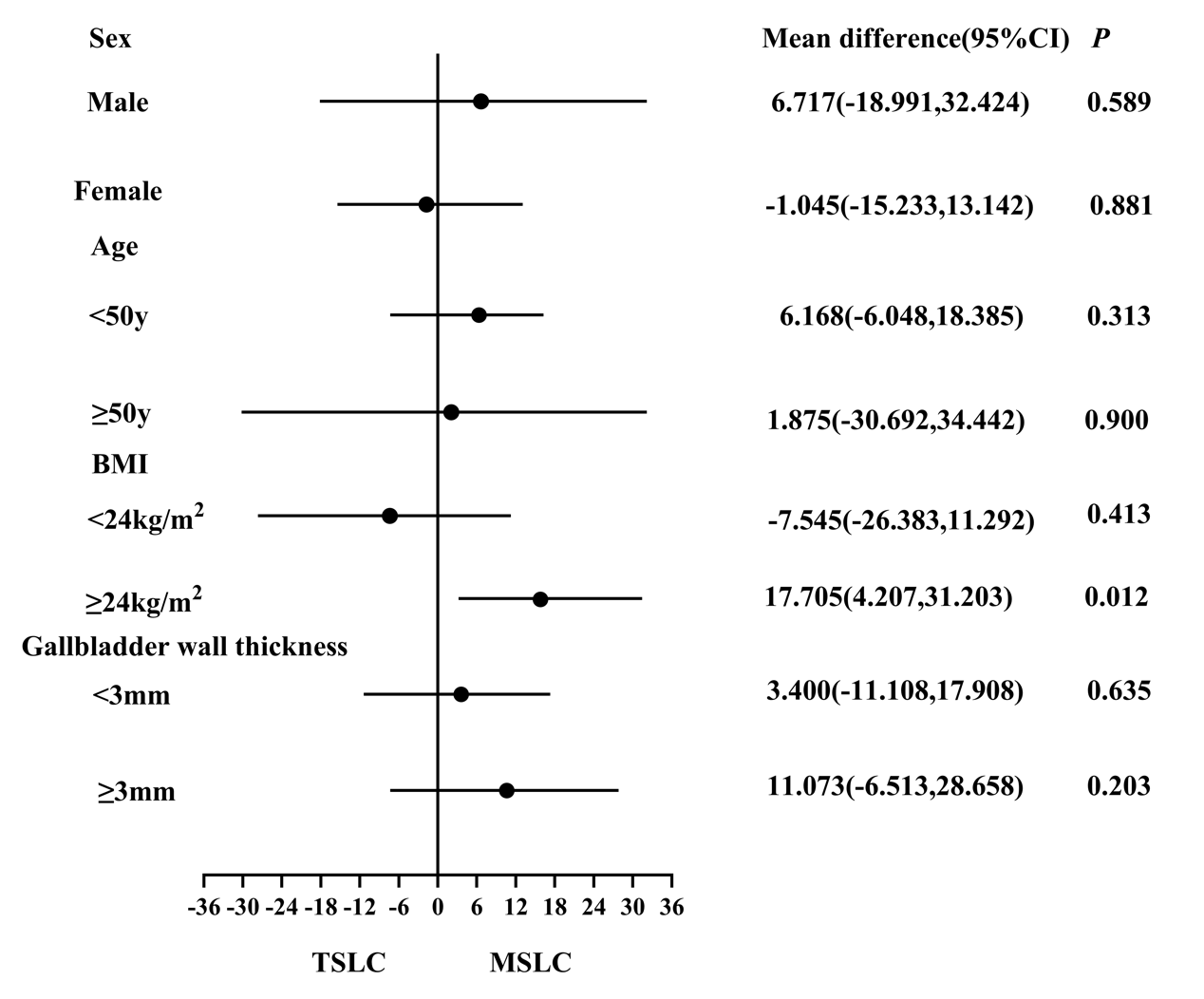

Supplement: Supplemental Information 1 [file peerj-14-20807-s001.zip › Figure2/Figure2.png]

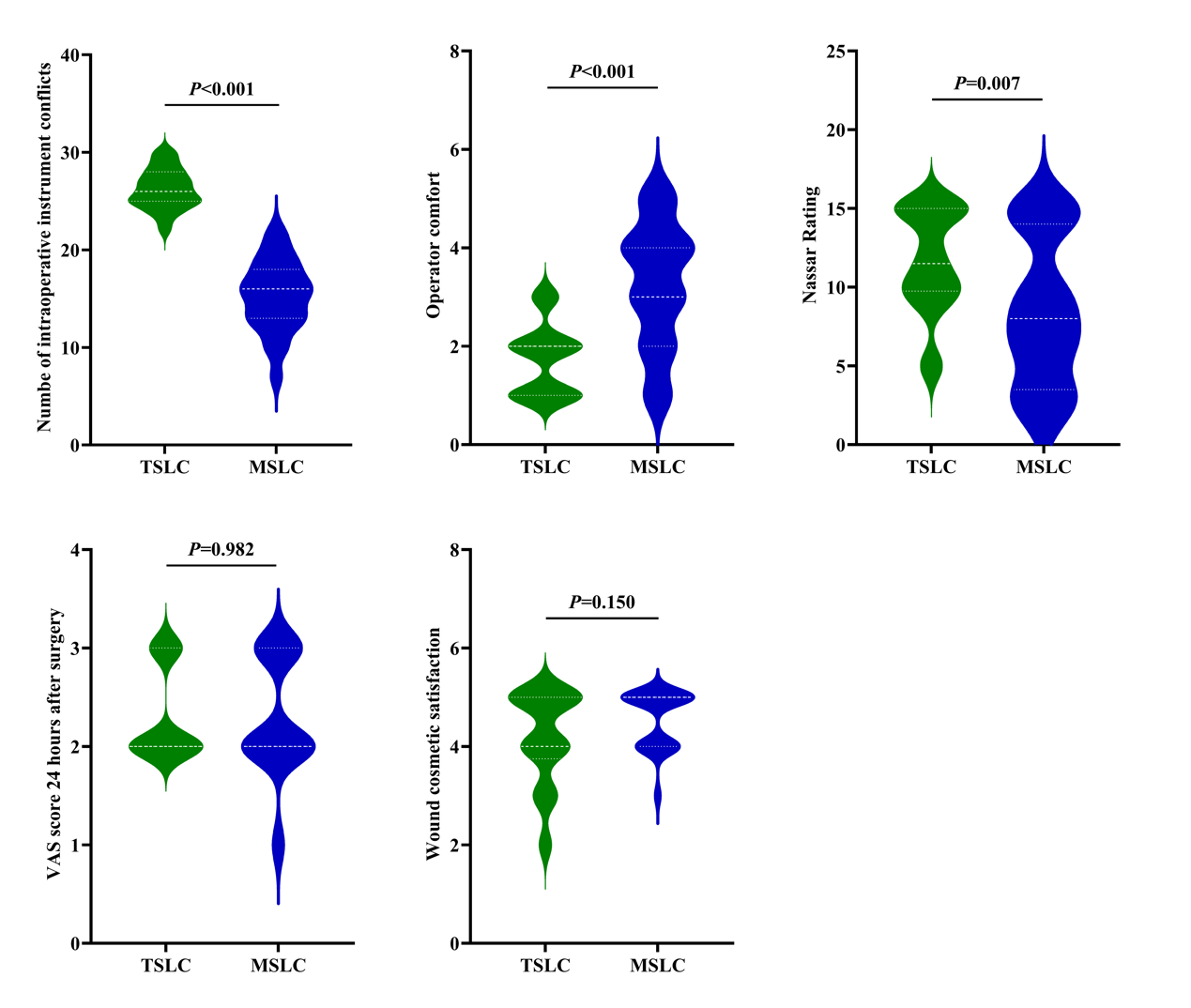

Supplement: Supplemental Information 1 [file peerj-14-20807-s001.zip › Figure3/Figure3.png]

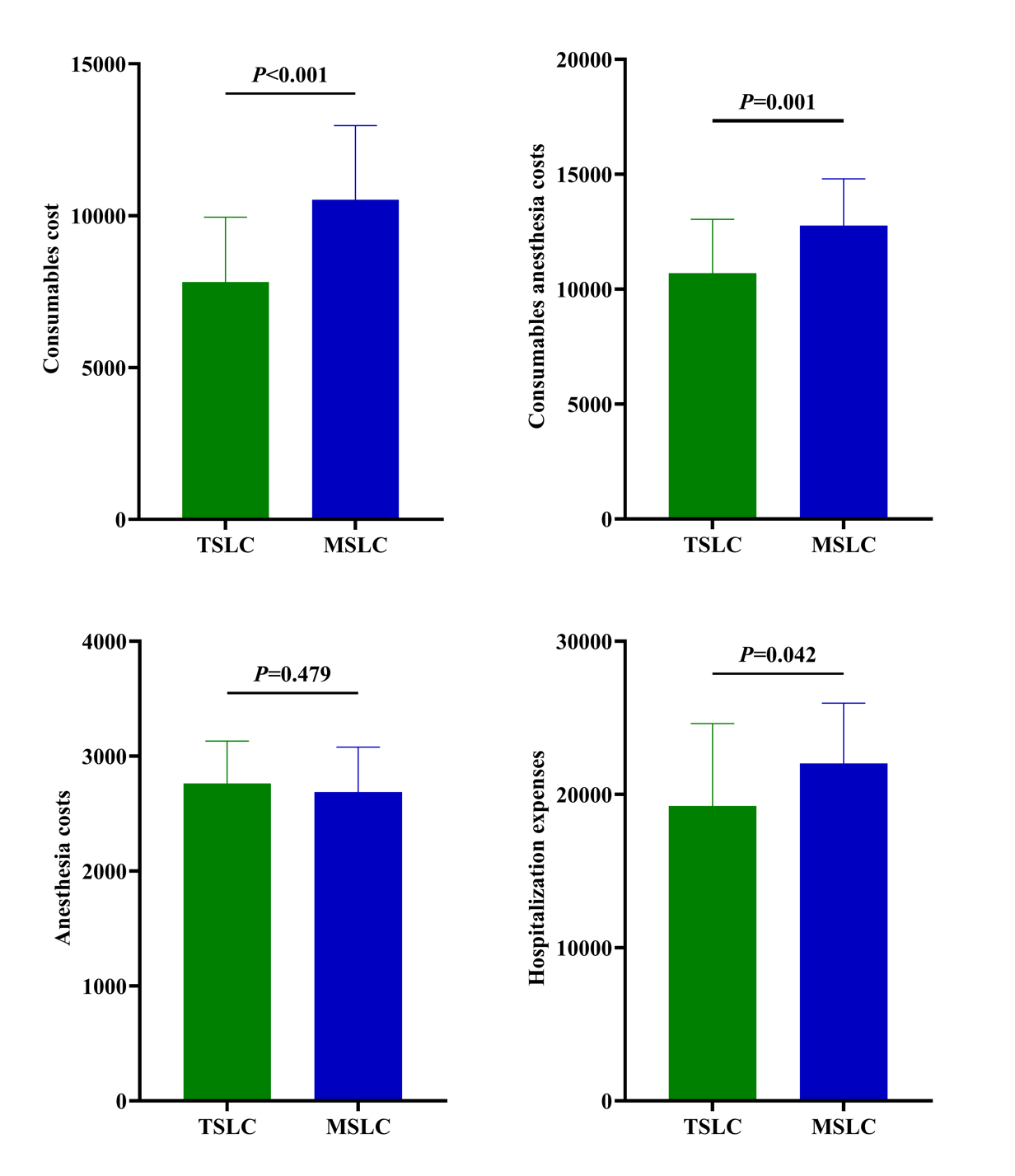

Supplement: Supplemental Information 1 [file peerj-14-20807-s001.zip › Figure4/Figure4.png]
